# *Cynoglossus semilaevis Rspo3* Regulates Embryo Development by Inhibiting the Wnt/β-Catenin Signaling Pathway

**DOI:** 10.3390/ijms19071915

**Published:** 2018-06-29

**Authors:** Jingjing Niu, Jian Guan, Rui Li, Xuemei Li, Jieming Zhai, Jie Qi, Yan He

**Affiliations:** 1 Key Laboratory of Marine Genetics and Breeding, Ocean University of China, Ministry of Education, Qingdao 266003, Shandong, China; nyjouc123@163.com (J.N.); guanjian@stu.ouc.edu.cn (J.G.); 17854252538@163.com (R.L.); xuemeili5750@163.com (X.L.); qijie@ouc.edu.cn (J.Q.); 2 Laizhou Mingbo Aquatic CO., Ltd., Laizhou 261418, Shandong, China; justdo123@163.com

**Keywords:** RT-PCR, epiboly, vascular development, cysteine-rich furin-like domain, tongue sole

## Abstract

*Cynoglossus semilaevis* is an important economic fish species and has long been cultivated in China. Since the completion of its genome and transcriptome sequencing, genes relating to *C. semilaevis* development have been extensively studied. *R-spondin 3* (*Rspo3*) is a member of the *R-spondin* family. It plays an important role in biological processes such as vascular development and oncogenesis. In this study, we cloned and characterized the expression patterns and functions of *C. semilaevis* *Rspo3*. Initial structural and phylogenetic analyses revealed a unique FU3 domain that exists only in ray-finned fish RSPO3. Subsequent embryonic expression profile analysis showed elevating expression of *Rspo3* from gastrulation to the formation of the eye lens, while, in tail bud embryos, *Rspo3* expression was significantly high in the diencephalon and mesencephalon. The overexpression of *C. semilaevis Rspo3* in *Danio rerio* embryos resulted in a shortened rostral–caudal axis, edema of the pericardial cavity, stubby yolk extension, and ecchymosis. Vascular anomalies were also observed, which is consistent with *Rspo3* role in vascular development. Drug treatment and a dual-luciferase reporter assay confirmed the inhibitory role of *C. semilaevis Rspo3* in *D. rerio* Wnt/β-catenin signaling pathway. We further concluded that the FU2, FU3, and TSP1 domains regulate the maternal Wnt/β-catenin signaling pathway, while the FU1 domain regulates the zygotic Wnt/β-catenin signaling pathway. This study enriches Rspo3 research in non-model animals and serves as the basis for further research into the interactions between *Rspo* and the Wnt/β-catenin signaling pathway.

## 1. Introduction

*Cynoglossus semilaevis* is one of the important economic fish species in China and has long been the subject of large-scale breeding projects. With the completion of its genome and transcriptome sequencing [1,2], *C. semilaevis* has undergone intensive studies mainly focusing on the molecular mechanisms of growth, sex determination, and immunity.

The *R-spondin* (*Rspo*) proteins are a family of secreted proteins that a share similar domain organization, with all four members characterized by a signal peptide at the N-terminus, followed by two or three furin-like cysteine-rich domains (FU), a thrombospondin-type 1 domain (TSP1), and a basic amino acid-rich C-terminal domain [3,4,5]. *Rspo1*–*4* are involved in embryonic development, cell proliferation, and tumorigenesis by activating the canonical Wnt/β-catenin pathway [6,7,8], and it has been demonstrated that the FU domains are responsible for such function [9].

*Rspo3*, the first known member of the *Rspo* family, was identified in a high-throughput sequencing study of a human fetal brain cDNA library [10] and was later studied in mouse [11,12,13], *Xenopus laevis* [14], *Danio rerio* [5], and other model species. RSPO3 plays a regulatory role by activating the Wnt/β-catenin signaling pathway. Moreover, RSPO3 is capable of inducing the non-canonical Wnt/PCP pathway by binding syndecan 4 (*Sdc4*) [15]. Though RSPO role as an agonist of the Wnt pathway has been widely recognized, there is also contradicting evidence showing that RSPO3 inhibits the Wnt/β-catenin signaling pathway in zebrafish embryos [5].

The functional study of *Rspo3* has mainly focused on its role in development. *Rspo3* is prominently expressed in blood-forming organs, and its deficiency leads to lethal vessel remodeling defects in mouse embryos [16]. *Rspo3* could also promote vascular development in *X. laevis* by upregulating the expression of VEGF through the activation of the Wnt/β-catenin signaling pathway. Since inhibiting the Wnt/β-catenin signaling pathway could strengthen hematopoiesis, *Rspo3* plays a role in keeping a balance between angiogenesis and hematopoiesis [14]. Moreover, a recent research shows that *Rspo3* is a crucial regulator of coronary artery formation in the developing heart [17]. Further research indicates that *Rspo3* can also maintain adult vessels. Overexpression of *Rspo3* tightens endothelium junctions and prevents vascular leakage, thereby mitigating the inflammatory events and associated tissue damage in a mouse model of mesenteric ischemia–reperfusion [18]. In addition, *Rspo3* is implied in limb, heart, and liver development [19,20,21].

Recently, the role of *Rspo3* in oncogenesis has also become a hotspot. *Rspo3* was identified as a protooncogene in a high-throughput study of retrovirus-induced mutant screening [22]. Later, *Rspo3* was found to be correlated to childhood leukemia, secondary thyroid carcinoma, and colon cancer [23,24]. In addition, a recent study indicates that endogenous *Rspo2* and *Rspo3* chromosome rearrangements can initiate and maintain tumor development [25].

More potential biological functions of *Rspo3* were recognized with the expansion of genome-wide association studies (GWAS). These studies suggest that *Rspo3* is involved in skeletal development and maintenance [26,27,28], fat distribution and metabolism [29,30,31], and expression of quantitative trait loci for human telomerase [32].

To date, most studies of *Rspo3* have been conducted in model organisms, but its role in non-model animals such as *C. semilaevis* has been poorly studied. In this research, we characterized the temporal and spatial expression profiles of *Rspo3* in the developmental stages of *C. semilaevis* and studied its function by overexpressing *C. semilaevis Rspo3* mRNA in zebrafish embryos. Furthermore, we studied the effect of *C. semilaevis Rspo3* on the Wnt/β-catenin signaling pathway and explored the functions of different protein domains. This research is a detailed study of *Rspo3* in non-model animals and provides insight for future *C. semilaevis* breeding.

## 2. Results

### 2.1. Cloning and Sequence Structure Analysis

**Figure 1:**
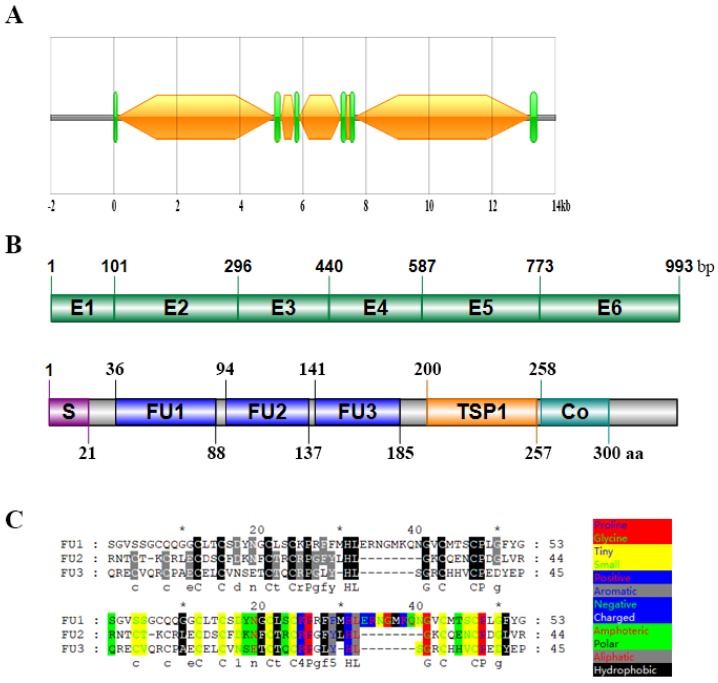
Sequence and structure of *Cynoglossus semilaevis Rspo3*/RSPO3. (**A**) Gene structure of *Rspo3*. Exons are shown in green quadrangles, whereas introns are shown in orange hexagons; (**B**) Above: exon composition of *C. semilaevis Rspo3* coding sequences (CDS). Below: conserved domains of *C. semilaevis* RSPO3. E: exon; S: leading signal peptide; FU: cysteine-rich furin-like domain; TSP1: thrombospondin-type1 domain; Co: coiled coil region; (**C**) Sequence and physicochemical analysis of three FU domains. Above: amino acids that are conserved in the three domains are shown in black, whereas those that are conserved in two domains are shown in grey. Below: different physicochemical properties are marked in different colors.

**Figure 2:**
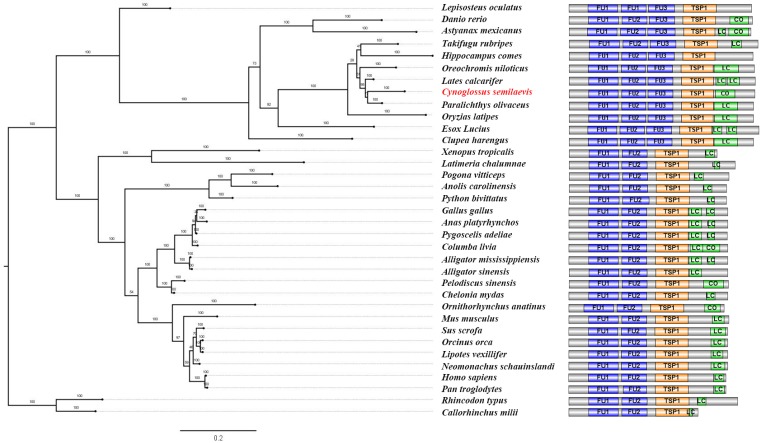
Phylogenetic and protein structure analysis of *C. semilaevis* and other vertebrate *Rspo3*/RSPO3 orthologs. The phylogenetic tree was constructed by MrBayes software, the lengths of the branches represent genetic distances, and bootstrap percentages are shown as numbers on the branches. The accession numbers are shown in the methods section. The protein structure is shown following the corresponding phylogenetic branch. FU: cysteine-rich furin-like domain; TSP1: thrombospondin-type1 domain; LC: low compositional complexity; Co: coiled coil region.

We cloned the *C. semilaevis Rspo3* gene by TA cloning and determined its gene structure by searching the NCBI database. *C. semilaevis Rspo3* gene contains six exons and five introns (Figure 1A,B). A 993bp open reading frame (ORF) was obtained, which encodes a polypeptide comprised of 330 amino acids (Figure 1B). The polypeptide contains the typical domains of RSPO proteins, namely, a leading signal peptide at the N-terminus, three cysteine-rich furin-like domains (FU1, FU2, and FU3), a thrombospondin type I repeat domain (TSP1), and a coiled coil region (Co) at the C-terminus (Figure 1B). Especially, the FU3 domain is a unique structural feature, which is undetected in RSPO3 of mammals, birds, reptiles, amphibians, and even Chondrichthyes, as we found (Figure 2), as well as in other proteins of the *C. semilaevis* RSPO family. Furthermore, FU3 is closer to the FU2 domain in terms of sequence conservation and physicochemical properties. The FU1 domain has an additional segment peptide, which contains eight miscellaneous amino acids (Figure 1C).

### 2.2. Phylogenetic Analysis

To elucidate the evolutionary history of the FU3 domain, a phylogenetic tree and a domain structure chart were constructed based on the amino acid sequences of RSPO3 from 35 species (Figure 2). Major vertebrate groups (cartilaginous fish, ray-finned fish, tetrapods) were roughly recovered, and this is supporting evidence for our correct ortholog identification. The phylogenetic analysis demonstrated that the RSPO3 orthologs of ray-finned fish were clustered into a clade, and those of tetrapods were clustered into another clade. All orthologs in the former clade have three FU domains, and all those in the latter clade have two FU domains. Ray-finned fish *Rspo3* orthologs encode an additional FU domain (FU3) and display a faster evolutionary rate.

### 2.3. Embryonic Expression Pattern

**Figure 3:**
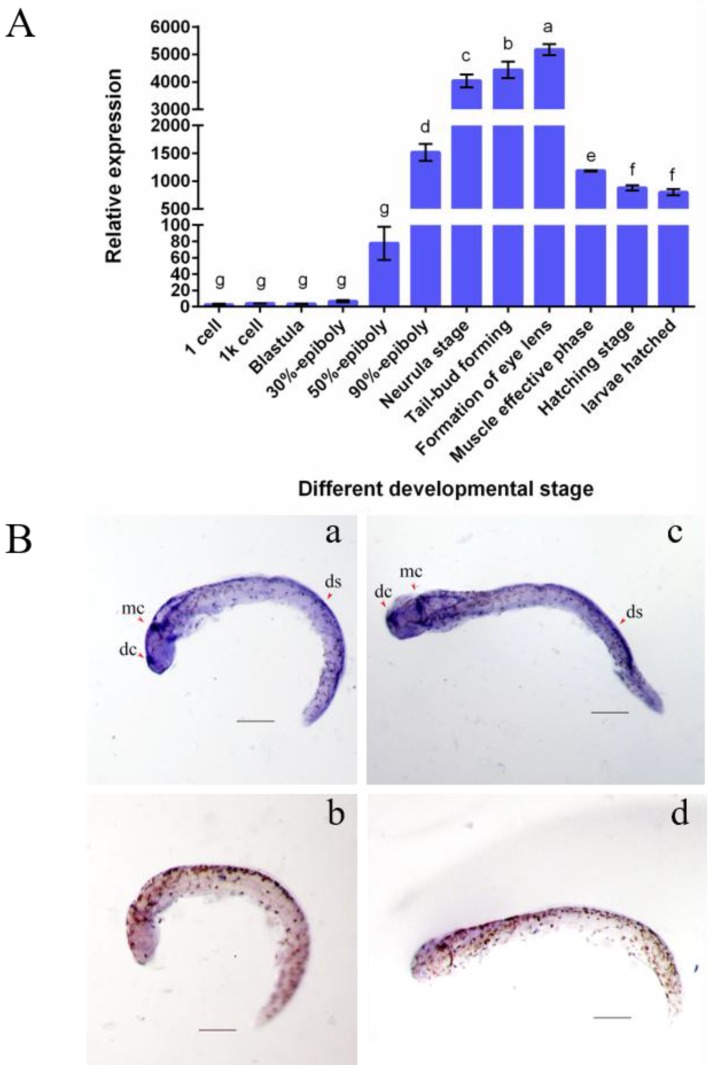
The spatiotemporal expression pattern of *C. semilaevis Rspo3*. (**A**) Expression profiles of *Rspo3* mRNA at different embryonic developmental stages. B2M is the reference gene. The data are shown as mean ± standard deviation (SD) (number of replicates, *n* = 3; number of total samples analyzed in each group, N = 90). Values with different superscripts indicate different statistical significance (*p* < 0.05); (**B**) Whole-mount in situ hybridization (WISH) analysis of *Rspo3* mRNA at tail bud stage. The embryos are shown in lateral (a and b) and quarter (c and d) views, with anterior to the left. Scale bars = 200 μm; a and c correspond to anti-sense probes, b and d to sense probes. dc: diencephalon; mc: metencephalon; ds: dorsal stripe.

RT-PCR analysis showed that *C. semilaevis Rspo3* mRNA was weakly expressed in the early developmental stages before 50%-epiboly and was highly expressed in the subsequent stages (Figure 3A). The amount of transcripts continued to rise from 50%-epiboly to the formation of the eye lens and then dramatically declined in the muscle-effective phase, entering a downtrend thereafter. To investigate the spatial expression pattern of *Rspo3* in *C. semilaevis*, whole-mount in situ hybridization (WISH) was carried out in tail bud-forming-stage embryos, and strong antisense signals were observed in the diencephalon, metencephalon, and dorsal stripe (Figure 3B).

### 2.4. The Impact of C. semilaevis Rspo3 Overexpression on the Development of D. rerio Embryos

**Figure 4:**
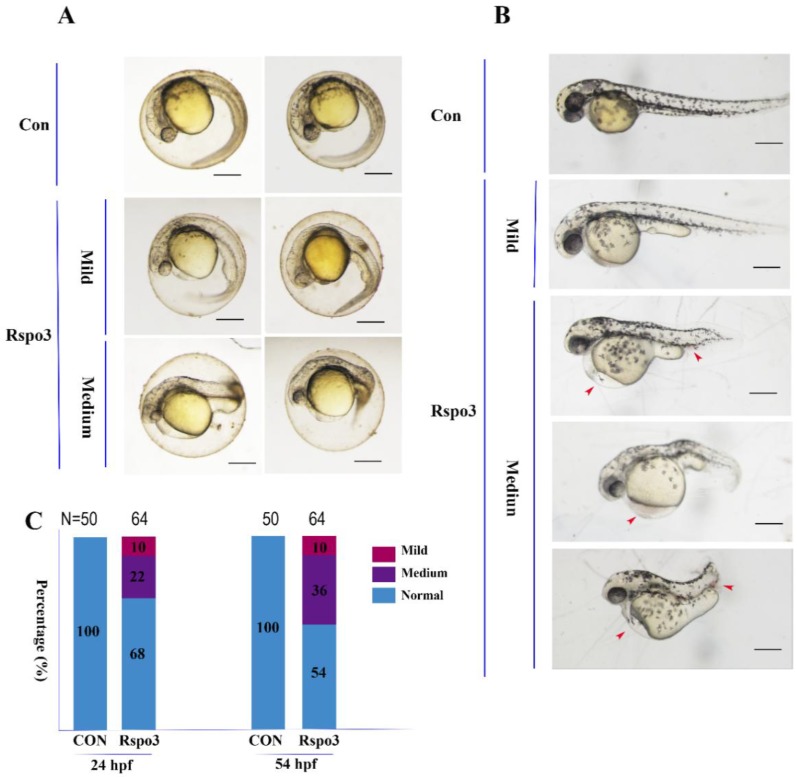
Overexpression of *C. semilaevis Rspo3* affects *Danio rerio* embryo development. An amount of 1000 pg/embryo of *C. semilaevis Rspo3* mRNA was injected into AB strain *D. rerio* embryos at 1–4 cell stages. All embryos are shown with anterior to the left. Scale bar = 200 μm. (**A**) Classification of phenotypes at 24 h post-fertilization (hpf); (**B**) Classification of phenotypes at 54 hpf. The red arrowheads indicate ecchymosis; (**C**) The percentages of embryos in each category as shown in (**A**) and (**B**); N is the number of total samples analyzed in each group.

To investigate its function in embryonic development, the capped mRNA of *C. semilaevis Rspo3* was injected into *D. rerio* 1–4 cell embryos. The overexpression of *C. semilaevis Rspo3* resulted in different degrees of malformation, with the phenotypic spectrum including shortened rostral–caudal axis, edema of the pericardial cavity, stubby yolk extension (Figure 4A,B), and ecchymosis in the pericardial cavity or the tail (Figure 4B). The deformation rate of 24 h post-fertilization (hpf) embryos was 33%, and that of 54 hpf embryos was 46% (Figure 4C).

**Figure 5:**
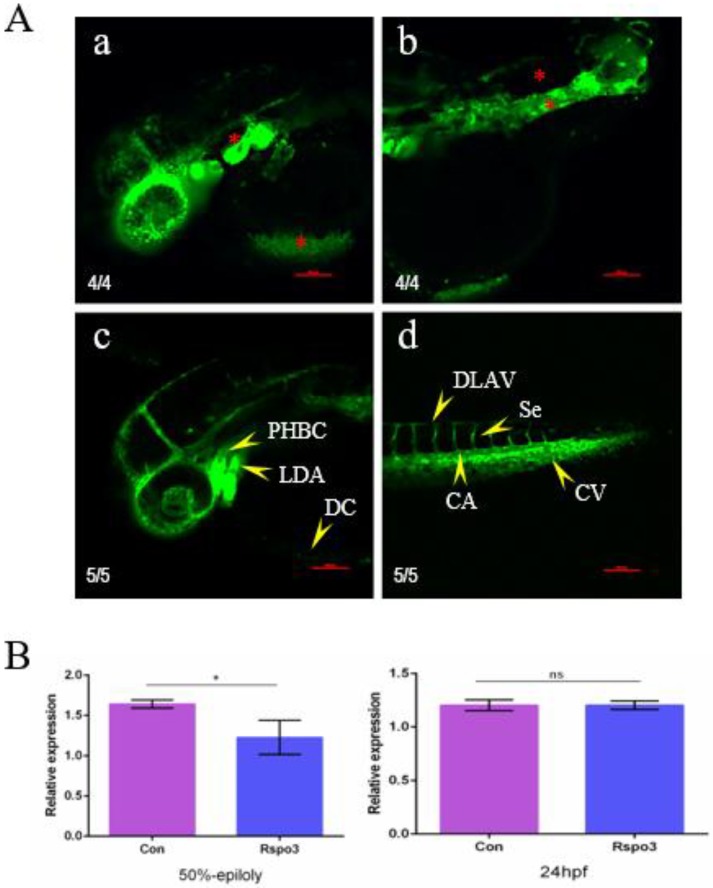
Overexpression of *C. semilaevis Rspo3* affects angiogenesis in *D. rerio* embryos. (**A**) Vascular abnormalities induced by overexpression of *C. semilaevis Rspo3* mRNA at 36 hpf; a and b show the most heavily impacted transgenic (*flila*: EGFP) strain *D. rerio* embryos. Each embryo was injected with 700 pg of *C. semilaevis Rspo3* mRNA at 1–4 cell stages; c and d are the control group without injection. The yellow arrowheads indicate different types of vessels, whereas the red asterisks point out the abnormalities in the experimental group. All panels show the embryos oriented with anterior to the left. Scale bar = 100 μm. LDA: lateral dorsal aorta, CCV: common cardinal vein, Se: intersegmental vessel, CV: caudal vein, CA: caudal aorta; (**B**) Expression analysis of VEGFa at the 50%-epiboly stage and 24 hpf. *β-actin* is the reference gene. The data are shown as mean ± SD (*n* = 3, N = 30). Values with different superscripts or an asterisk indicate different statistical significance (* *p* < 0.05; ns *p* > 0.05).

Ecchymosis suggests that vascular development might have been affected, which was further substantiated by the observation of vascular anomalies and of the downregulation of VEGFa expression in 50%-epipoly embryos (Figure 5). Among all blood vessels, optic vein (OV), lateral dorsal aorta (LDA), common cardinal vein (CCV), intersegmental vessel (Se), caudal vein (CV), and caudal aorta (CA) were the most seriously affected by the overexpression of *C. semilaevis Rspo3* in transgenic strain (*flila*:EGFP) zebrafish embryos, with anomalous and undifferentiated LDA, absent Se, chaotic CV and CA (Figure 5A). The expression of VEGFa was significantly downregulated in the presence of excess *Rspo3* in 50%-epiboly embryos but was not significantly affected in 24 hpf embryos (Figure 5B). The developmental disorders indicated that gastrulation might have been disrupted in mRNA-injected zebrafish embryos.

### 2.5. C. semilaevis Rspo3 Plays a Role in Gastrulation by Regulating the Wnt/β-Catenin Signaling Pathway

We chose *no tail* (*ntl*), a marker gene of mesoderm, to assess the impact of *C. semilaevis Rspo3* on the Wnt/β-catenin signaling pathway and gastrulation. Three groups of zebrafish embryos were incubated with the Wnt/β-catenin signaling pathway activator 6-bromoindirubin-3′-oxime (BIO) or with the inhibitor XAV-939 or were microinjected with capped *C. semilaevis Rspo3* mRNA, and their *ntl* expression levels were analyzed by RT-PCR and WISH.

**Figure 6:**
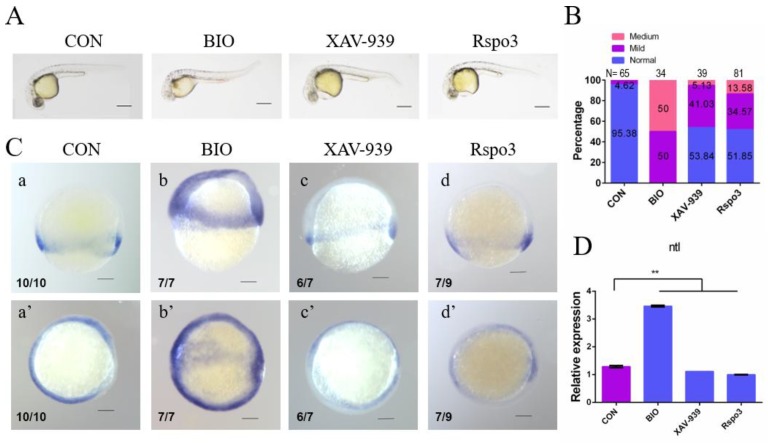
*C. semilaevis Rspo3* affects mesoderm formation by regulating the Wnt/β-catenin signaling pathway. (**A**) AB strain *D. rerio* 24 hpf embryos after treatment with 0.15 μM BIO or 1.67 μM XAV-939, or microinjection with 1000 pg *C. semilaevis Rspo3* mRNA per embryo. All embryos are shown oriented with anterior to the left. Scale bar = 200 μm; (**B**) Abnormality rates of embryos in each group as shown in (**A**), N is the number of total samples analyzed in each group; (**C**) WISH analysis of *ntl* at the gastrula stage in each group; a–d are lateral views, whereas a’–d’ are quarter views. Scale bar = 200 μm; (**D**) RT-PCR analysis of *ntl* at the gastrula stage in each group. *β-actin* is the reference gene. The data are shown as mean ± SD (*n* = 3, N = 30). The superscript indicates statistical significance (** *p* < 0.01).

We observed that, in terms of *ntl* expression patterns and phenotype, the mRNA-injected embryos were closer to XAV-939-treated embryos than to embryos treated with BIO. The overall expression of *ntl* was significantly downregulated during the epiboly phase in both mRNA-injected and XAV-939-treated embryos but was upregulated in BIO-treated embryos (Figure 6D). Moreover, the expression of *ntl* in both the mesoderm and the ectoderm increased after BIO treatment, while it decreased in the mesoderm of mRNA-injected or XAV-939-treated groups, especially in the ventral mesoderm (Figure 6C). The embryos of all three groups displayed a similar proportion of mild pericardial edema, whereas medium anomalies were much more prevalent in BIO-treated embryos (Figure 6B). Such similarities between *C. semilaevis Rspo3* and XAV-939 indicate the possibility that the former regulates gastrulation by serving as an inhibitor of the zebrafish Wnt/β-catenin signaling pathway.

### 2.6. C. semilaevis Rspo3 Suppresses the Wnt/β-Catenin Signaling Pathway in D. rerio Embryos

**Figure 7:**
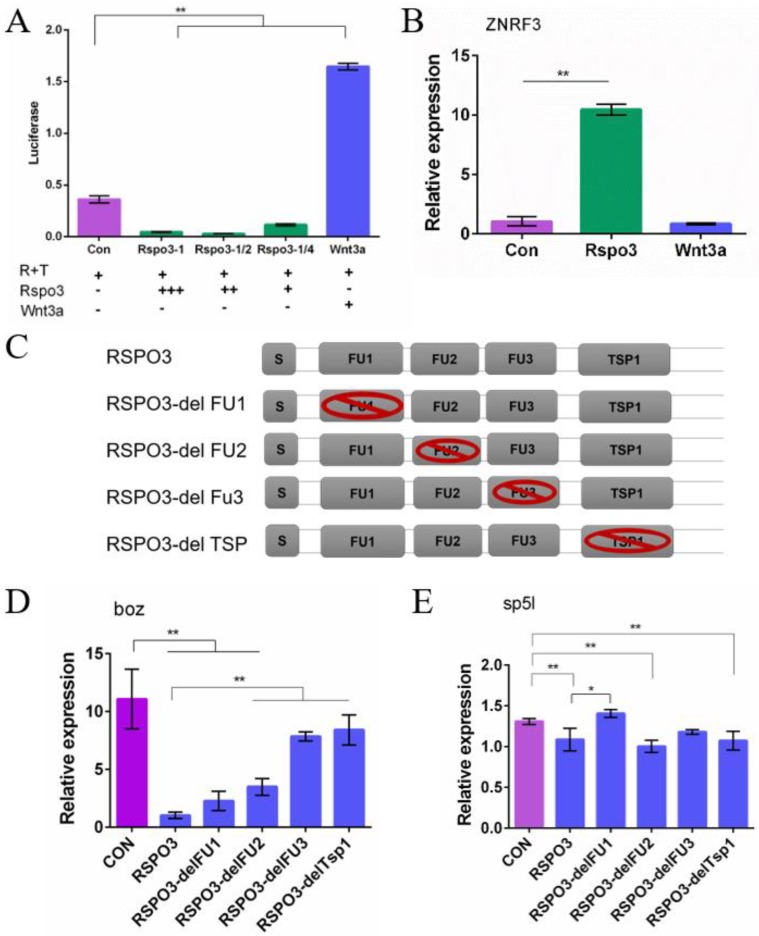
*C. semilaevis Rspo3* inhibits the Wnt/β-catenin signaling pathway in *D. rerio* embryos. (**A**) The dual-luciferase reporter assay shows that *C. semilaevis Rspo3* inhibits Wnt/β-catenin reporter activities. AB strain *D. rerio* embryos at 1–4cell stage were injected with 200 pg TOPFlash plasmid and 40 pg Renilla plasmid together with 1400 pg, 700 pg, 350 pg *C. semilaevis Rspo3* mRNA or 20 pg zebrafish Wnt3a mRNA each. *β-actin* is the reference gene. The data are shown as mean ± SD (*n* = 3, N = 90). The superscripts indicate statistical significance (** *p* < 0.01); (**B**) RT-PCR analysis of ZNRF3 at 50%-epiboly stage. AB *D. rerio* embryos at 1–4 cell stage were injected with 700 pg *C. semilaevis Rspo3* mRNA or 20 pg *D. rerio Wnt3a* mRNA. The data are shown as mean ± SD (*n* = 3, N = 30). The superscripts indicate statistical significance (** *p* < 0.01); (**C**) The core structure composition of each domain-deleted RSPO3 plasmid. (**D**,**E**) RT-PCR analysis of *boz* and *sp5l* at the 50%-epiboly stage. AB *D. rerio* embryos at the 1–4 cell stage were injected with 500 pg *C. semilaevis Rspo3* mRNA or domain-deleted *C. semilaevis Rspo3* mRNA. The data are shown as mean ± SD (*n* = 3, N = 30). Values with different superscripts indicate different statistical significance (* *p* < 0.05; ** *p* < 0.01).

A dual-luciferase reporter assay was carried out to further substantiate the role of *Rspo3*. Luciferase activity in embryos microinjected with *C. semilaevis Rspo3* mRNA was significantly downregulated, while that in embryos microinjected with *D. rerio* Wnt3a mRNA was significantly upregulated (Figure 7A). Moreover, RT-PCR showed that the expression of *boz*, a target gene of *D. rerio* maternal β-catenin, was significantly downregulated by overexpressing *C. semilaevis Rspo3* mRNA but was significantly upregulated by BIO treatment. Unfortunately, WISH results were not as obvious as those of RT-PCR and only showed a slight change after mRNA injection. To understand the inhibitory role of *C. semilaevis Rspo3* in the Wnt/β-catenin pathway, we characterized the expression of ZNRF3, an RSPO receptor, in zebrafish embryos overexpressing *Rspo3* and Wnt3a, respectively. We demonstrated that ZNRF3 expression was significantly upregulated after *Rspo3* overexpression but did not show any changes after Wnt3a overexpression (Figure 7B).

In order to study the roles of different *C. semilaevis* RSPO3 domains in regulating the Wnt/β-catenin signaling pathway, four types of domain-deletion plasmids (P*^RSPO3^*^-delFU1^, P*^RSPO3^*^-delFU2^, P*^RSPO3^*^-delFU3^, P*^RSPO3^*^-delTSP1^) were constructed by seamless cloning (Figure 7C). Each one of the five groups of 50%-epiboly *D. rerio* embryos were microinjected with one type of domain-deletion *Rspo3* mRNA or wild-type *Rspo3* mRNA. The samples were analyzed to detect the expression of the direct maternal β-catenin target gene *boz* and the zygotic Wnt/β-catenin signaling target gene *sp5l* [5,33]. The expression of both *boz* and *sp5l* was significantly downregulated after injecting wild-type *Rspo3* mRNA (Figure 7D,E). However, FU3- and TSP1-deleted *Rspo3* mRNA failed to induce a significant decrease in *boz* expression, while, though both FU1- and FU2-deleted *Rspo3* mRNA resulted in a significant downregulation of *boz* expression, only the former displayed no significant differences compared with the effects of the wild-type plasmid, indicating that only the FU1 domain is dispensable for inhibiting the maternal Wnt/β-catenin signaling pathway. In comparison with *boz*, the expression of *sp5l* was not significantly changed by FU1- and FU3-deleted *Rspo3* mRNA, while only the former displayed a significant difference compared to that of the wild-type plasmid, indicating that it inhibited the zygotic Wnt/β-catenin signaling pathway. Taken together, these results indicate that the FU1 domain is the major inhibitor of the zygotic Wnt/β-catenin signaling pathway, while the other three domains are responsible for inhibiting the maternal Wnt/β-catenin signaling pathway.

## 3. Discussion

In this study, we characterized the ORF sequence of *C. semilaevis Rspo3*, which is 993 bp long and encodes a peptide of 330 amino acids. Though most RSPO proteins contain only two FU domains, RSPO3 of *C. semilaevis* and other ray-finned fish species contain three FU domains [5]. A phylogenetic analysis also showed that *Rspo3* genes with three FU domains have a faster evolutionary rate than those with two FU domains, and FU3 is closer to the FU2 domain compared with the FU1 domain in terms of sequence conservation and physicochemical properties.

The analysis of the embryo expression profiles showed elevating mRNA expression in *C. semilaevis* from the gastrula stage until the formation of the eye lens and a relatively low expression level thereafter. There was a significant elevation of *Rspo3* expression from the 50%-epiboly to the 90%-epiboly stage, which coincides with the formation of three germ layers. The formation of three germ layers is one of the most important events during gastrulation and is critical to organ and tissue fate map determination [34]. This indicates that *C. semilaevis Rspo3* might play a role in regulating the formation and patterning of the germ layers. The temporal expression pattern of *C. semilaevis Rspo3* is analogous to that of *D. rerio* and is different from that of mouse and *Xenopus*. The expression of *D. rerio* *Rspo3* mRNA was initially detected in fertilized eggs and remarkably increased at 9 hpf (90%-epiboly stage) [5]. In contrast, the expression of *Rspo3* was initially detected at the gastrulation stage in *Xenopus* [6] and in the primitive streak at embryonic day 7.5 (E7.5) in mouse [13]. However, *Rspo3* is expressed at a relative high level during gastrulation in all these species, which indicates that the role of *Rspo3* as a regulator of gastrulation is highly conserved.

After gastrulation, the expression of *C. semilaevis Rspo3* mRNA was detected in tail bud embryos. Positive signals were detected in the diencephalon and mesencephalon. The spatial expression patterns of *Rspo3* displayed both similarities and differences among the aforementioned species. *D. rerio Rspo3* displayed a tissue specific expression pattern after 12 hpf, with strong signals detected in brain (including telencephalon, diencephalon, metencephalon, rhomb-encephalon) and other tissues [5]. Mouse *Rspo3* mRNA was detected in the posterior primitive streak and allantois at E8.0 and in the forebrain, dorsal neural tube, migrating neural crest cells, limb bud, and developmental heart from E9.0 to E10.5 [13,14,35]. *X. laevis Rspo3* mRNA was detected in the neural plate at stage 17 and in the forebrain, branchial arches, pronephric sinus, dorsal neural tube, dorsal plate, ventral blood island, and tail-bud mesoderm at stage 28 [14]. The conservative expression of *Rspo3* in the brain among these species suggests that *Rspo3* is involved in the development of the central nervous system. Regrettably, we did not get more spatial expression information for *C. semilaevis* because only tail bud embryos were analyzed by WISH.

Because of the infeasibility of microinjection in *C. semilaevis* embryos and considering the similarity of protein structure and expression patterns between *C. semilaevis* and *D. rerio Rspo3*, a gain-of-function analysis was conducted by overexpressing *C. semilaevis Rspo3* mRNA in *D. rerio* embryos. The forced expression of *Rspo3* caused phenotypic malformations in *D. rerio*, including a shorter axis, edema of the pericardial cavity, stubby yolk extension, and ecchymosis in the pericardial cavity or tail. Vascular anomalies were identified by fluorescence and indicated by the decreased of VEGFa in 50%-epiboly embryos. The mechanisms of such abnormal development were studied by using molecular markers. Since gastrulation is regulated by the Wnt/β-catenin signaling pathway, and previous studies have implied the involvement of *Rspo3* in regulating the Wnt/β-catenin signaling pathway [3,4,5,9], we speculated that *C. semilaevis Rspo3* could also interact with this pathway in zebrafish. The expression of *ntl* in *D. rerio* was downregulated by overexpressing *C. semilaevis Rspo3* or treating with XAV-939. This indicated that *C. semilaevis Rspo3* regulates the formation and patterning of mesoderm via regulating the Wnt/β-catenin signaling pathway.

*Rspo3* expression is related to vasculogenesis and angiogenesis in both *Xenopus* and mouse. *Xenopus Rspo3* is expressed in the dorsal lateral plate and ventral blood islands [14], while mouse *Rspo3* is expressed in the posterior primitive streak, allantois, and blood vessels [14,35]. All these sites are related to vasculogenesis and angiogenesis. It was further confirmed that *Rspo3* regulates the balance between blood and endothelial differentiation by triggering Wnt/β-catenin signaling to activate the immediate early target gene VEGF [14]. Consequently, the ecchymosis observed in the pericardial cavity or tail in seriously affected *D. rerio* embryos by overexpressing *C. semilaevis Rspo3* may result from the downregulation of VEGFa. This speculation was substantiated by observations in transgenic strain (*flila*: EGFP) zebrafish embryos. Based on the above results, the abnormal phenotype and downregulation of VEGFa suggest that *C. semilaevis Rspo3* inhibits the Wnt/β-catenin signaling pathway.

Although previous research conducted in *Xenopus* and other vertebrates revealed an activatory role of *Rspo* in regulating the Wnt/β-catenin signaling pathway [4,9,12,36,37], there is also contradicting evidence showing that *Rspo3* inhibits the Wnt/β-catenin signaling pathway in *D. rerio* embryos [5]. We employed a dual-luciferase reporter assay to further substantiate our conclusions in *C. semilaevis*. Contrary to Wnt3a, *Rspo3* significantly reduced TOPFlash reporter expression induced by endogenous Wnt/β-catenin signaling pathway. In addition, the expression of the maternal β-catenin target gene *boz* and zygotic Wnt/β-catenin signaling target gene *sp5l* were downregulated at 50%-epiboly by overexpressing *C. semilaevis Rspo3*, supporting the conclusion that *C. semilaevis Rspo3* inhibits the Wnt/β-catenin signaling pathway. Taken together, our results suggest that *C. semilaevis Rspo3* suppresses Wnt/β-catenin signaling pathway in *D. rerio*.

In *D. rerio* embryos, maternal β-catenin accumulates in dorsal margin blastomeres and establishes dorsal patterning by activating the expression of *boz* after the mid-blastula transition, while zygotic Wnt/β-catenin signaling is essential for the specification of the ventral and posterior fates [33,38]. Though the expression of *boz* is not affected significantly by overexpressing *Rspo3* in *D. rerio* at the sphere stage [5], our study showed that it was downregulated at the 50%-epiboly stage by overexpressing *C. semilaevis Rspo3* in *D. rerio* embryos, which indicates that the zygotic Wnt/β-catenin signaling pathway is functional at the 50%-epiboly stage but not at the sphere stage. Regardless of the cause, the downregulation of *boz* and *sp5l* indicates that *C. semilaevis Rspo3* inhibits the Wnt/β-catenin signaling pathway in zebrafish.

We studied the expression of ZNRF3, one of the RSPO receptors, in order to explore the mechanisms of the interaction between *C. semilaevis Rspo3* and the Wnt/β-catenin signaling pathway. LGR4-6 and ZNRF3/RNF43 have been identified as RSPO receptors [37,39,40,41,42]. LGR4-6 are G protein-coupled receptors with leucine-rich repeats, whose binding effects have been confirmed by a series of experiments [37,39,40,43,44], and it has been shown that RSPO binds to LGR4-6 via the FU domain [45]. However, a recent study discovered that RSPO2 and RSPO3 can activate the Wnt/β-catenin signaling pathway in the absence of LGR4-6 [46]. ZNRF3 and RNF3, another type of RSPO receptors, are E3 ubiquitin-ligase enzymes that can downregulate the Wnt signaling pathway by promoting ubiquitination and degradation of Wnt receptors. It was found that RSPO, LGR4-6, ZNRF3, and RNF43 can form a ternary complex to eliminate the FZD–LRP complex from the membrane [41,42]. Our study shows that the expression of ZNRF3 in 50%-epiboly zebrafish embryos was significantly upregulated by overexpressing *C. semilaevis Rspo3*. This provides a new perspective to understand the inhibitory role of *C. semilaevis Rspo3* in regulating the Wnt/β-catenin signaling pathway.

We further constructed different types of domain-deletion plasmids to explore the contribution of different domains regulating the Wnt/β-catenin signaling pathway. The expression of *boz* and *sp5l* in 50%-epiboly *D. rerio* embryos microinjected with the corresponding domain-deletion *Rspo3* mRNA was detected. We concluded that the FU2, FU3, and TSP1 domains are important for regulating the maternal Wnt/β-catenin signaling pathway, while the FU1 domain is important for regulating the zygotic Wnt/β-catenin signaling pathway. It was reported that RSPO1-2 can bind to RNF3 and ZNRF3 via the FU1 domain and to LGR via the FU2 domain and that the ability of binding ZNRF3/RNF3 differs significantly among RSPO1–4 [47,48]. A recent research demonstrated that the heparin sulfate proteoglycans (HSPGs) were essential in LGR-independent signaling [46]. Our study showed that different domains contributed differently to the inhibition of the Wnt//β-catenin signaling pathway, and *C. semilaevis Rspo3* regulated the maternal and zygotic Wnt/β-catenin signaling pathway depending on different domains.

However, the interactions between receptors and specific *C. semilaevis Rspo3* domains need to be studied further. In addition, phylogenetic and physicochemical analyses showed that the FU3 domain is closer to the FU2 domain, while the FU1 domain has an extra peptide containing eight consecutive and miscellaneous amino acids. Whether the structural and physicochemical differences among the FU domains play a role in regulating interactions with other proteins also needs further study.

In conclusion, the expression and function of *C. semilaevis Rspo3* were studied in this research. Our results enrich the study of *Rspo3* in non-model animals and provide insight for *C. semilaevis* breeding.

## 4. Methods

### 4.1. Samples

*C. semilaevis* fish and embryos were collected from Laizhou Mingbo Aquatic Co., Ltd., in Yantai, China. Tissues and organs, including brain, gill, heart, intestine, kidney, liver, muscle, spleen, and gonads were collected from three one-year-old male and female individuals for RNA extraction. Adult tissues and embryos for RNA extraction were stored in RNAwait (Solarbio), and embryos for WISH were stored in 4% PFA.

Zebrafish, 1–1.5-year-old, (wild-type AB strain and transgenic strain (*flila*: EGFP)) were cultured under a 14 h light–10 h dark cycle. Embryos were obtained by natural crossing, kept in Holtfreter buffer at 28.5 °C, and staged according to standards proposed by Kimmel et al. [34] The samples for RNA extraction were frozen in liquid nitrogen and stored at −80 °C.

All experimental protocols concerning animals were conducted in accordance with the regulations of the Ethical Committee of Experimental Animal Care, Ocean University of China (Permit Number: 11001).

### 4.2. Total RNA Extraction and cDNA Synthesis

Total RNA from adult tissues and embryos was extracted with Trizol Reagent (Invitrogen, Carlsbad, CA, USA) according to the manufacturer’s protocol. Adult tissue cDNA synthesis was performed with Reverse Transcriptase M-MLV Kit (TaKaRa, Dalian, China), while embryo cDNA was synthesized by PrimeScript™ RT reagent Kit with gDNA Eraser (Perfect Real Time) (TaKaRa). cDNA purity and concentration were determined by 1.5% agarose gel electrophoresis and UV spectrophotometry using NanoPhotometer Pearl (Implen, Schatzbogen, Germany).

### 4.3. Molecular Cloning

The ORF sequence of *C. semilaevis Rspo3* was amplified using the TaKaRa Ex-*Taq* PCR kit and ligated to vector of pMD18-T (Takara). The template was mixed adult tissue cDNA, the pair of primers consisted of CS-*Rspo3*-ORF-Fw and CS-*Rspo3*-ORF-Rv (Table 1). The adult tissue was dissected from three one-year-old female fish (average weight is 145 ± 10 g) and three one-year-old male fish (average weight is 100 ± 7 g).

### 4.4. Phylogenetic Analysis

Homologous amino acid sequences of *Rspo3* were retrieved from NCBI (available online: http://www.ncbi.nlm.gov) and Ensemble (available online: www.ensembl.org). The sequences cover 35 species, including *Homo sapiens* (NP_116173.2), *Mus musculus* (NP_082627.3), *Pan troglodytes* (XP_001166327.1), *Sus scrofa* (NP_001302585.1), *Orcinus orca* (XP_004263897.1), *Neomonachus schauinslandi* (XP_021549174.1), *Ornithorhynchus anatinus* (XP_007656158.1), *Gallus gallus* (AGG55029.1), *Columba livia* (XP_005507484.1), *Anas platyrhynchos* (XP_005009653.1), *Alligator mississippiensis* (XP_006272521.2), *Pygoscelis adeliae* (XP_009323912.1), *Pogona vitticeps* (XP_020646342.1), *Anolis carolinensis* (XP_008120556.1), *Python bivittatus* (XP_007421067.1), *Pelodiscus sinensis* (XP_006139494.1), *Alligator sinensis* (XP_006027792.1), *Chelonia mydas* (XP_007064459.1), *Xenopus tropicalis* (NP_001123245.1), *Rhincodon typus* (XP_020372626.1), *Callorhinchus milii (*XP_007897453.1), *Latimeria chalumnae* (XP_006006810.1), *Lipotes vexillifer* (XP_007448028.1), *Lepisosteus oculatus* (XP_015205296.1), *Danio rerio* (NP_001017358.1), *Takifugu rubripes* (XP_003966018.1), *Oryzias latipes* (NP_001239178.1), *Astyanax mexicanus* (XP_007251874.1), *Esox lucius* (XP_012994802.1), *Hippocampus comes* (XP_019750648.1), *Oreochromis niloticus* (ENSONIP00000009097), *Lates calcarifer* (XP_018540154.1), *Clupea harengus* (XP_012692909.1), *Paralichthys olivaceus* (local data), and *Cynoglossus semilaevis* (local data). A phylogenetic tree was constructed by MrByes software (V.3.2.2), mcmc = 200,000 generations [49]. The protein domains were predicted by SMART (available online: http://smart.embl-heidelberg.de) and NCBI CD-search (available online: http://www.ncbi.nlm.nih.gov/Structure/cdd/wrpsb.cgi), and the schematic diagram was constructed by the online tool IBS (available online: http://ibs.biocuckoo.org/online.php).

### 4.5. RT-PCR Analysis and WISH

RT-PCR was employed to analyze the mRNA expression of different target genes and was executed using SYBR^®^ Green I kit (Takara, Kagosima, Japan) on Roche Lightcycler 480. B2M was used as a reference gene in *C. semilaevis*, and *β-actin* was used as a reference gene in *D. rerio*. The primers containing “RT-PCR” in their names were used for RT-PCR (Table 1). The expression levels of different target genes were calculated using the 2-ΔΔ*C*t method. Each gene was amplified in triplicates, and the numeric data were presented as mean ± SD. Statistical significance was tested by one-way ANOVA using SPSS 20.0 (SPSS, Armonk, NY, USA) and was accepted when *p* < 0.05.

WISH was carried out as described previously [50]. The sense and antisense mRNA probes of different target genes were synthesized with DIG RNA Labeling Mix (Indianapolis, IN, USA). Two pairs of primers (CS-*Rspo3*-WISH-Fw/Rv and DR-*ntl*-WISH-Fw/Rv) were used for probe synthesis (Table 1). The results were photographed by AZ100 (Nikon, Tokyo, Japan).

### 4.6. mRNA Synthesis and Microinjection

Capped mRNA of different target genes was synthesized with mMESSAGE mMACHINE@T7 (Ambion, Foster City, CA, USA). The primers were CS-*Rspo3*-mRNA-Fw and CS-*Rspo3*-mRNA-Rv (Table 1). Microinjection was carried out on Harvard Apparatus PLI-100 (NatureGene, NV, USA) machine in one- to four-cell-stage embryos with 1 nl *Rspo3* mRNA for each embryo. Embryos not injected were the control group.

### 4.7. Luciferase Report Assay

Luciferase report assays were performed with the Dual-Luciferase^®^ Reporter Assay System (Promega, Madison, WI, USA), following the manufacturer’s instructions. TOPFlash was used as a report plasmid, and Renilla was used as a reference plasmid. *D. rerio* embryos at one- to four-cell stage were injected with a mixture of target gene mRNA, TOPFlash plasmid, and Renilla plasmid, and raised until shield stage. Three independent samples (each with 30 embryos) of each group were lysed, and the ratio of firefly luciferase to Renilla luciferase was used as relative luciferase activity.

### 4.8. Drug Treatment

The activator BIO and the inhibitor XAV-939 were used to regulate the Wnt/β-catenin signaling pathway. *D. rerio* embryos were incubated in corresponding solutions until phenotype observation and statistics or sampling for RNA extraction and WISH were performed.

### 4.9. Mutant Plasmids Construction

Wild-type (P^RSPO3^) and mutant plasmids (P^RSPO3-delFU1^, P^RSPO3-delFU2^, P^RSPO3-delFU3^, P^RSPO3-delTSP1^) were constructed to study the function of the protein domains. P^RSPO3^ was constructed by inserting *C. semilaevis Rspo3* ORF sequence into P^CS2+EGFP^ plasmid using *Eco*RI and *Bam*HI restriction sites. The pair of primers was CS-*Rspo3*-ORF-EC-Fw and CS-*Rspo3*-ORF-EC-Rv (Table 1). The mutant plasmids were constructed by seamless cloning. Four pairs of primers (CS-*Rspo3*-delFU1-Fw /Rv, CS-*Rspo3*-delFU2-Fw/Rv, CS-*Rspo3*-delFU3-Fw/Rv, CS-*Rspo3*-delTSP1-Fw/Rv; Table 1) were designed by the online tool IDT (available online: https://www.genomics.agilent.com/primerDesignProgram.jsp). Pfu DNA polymerase (ThermoFish Scientific, Shanghai, China) was used for PCR, and *Dpn*I (NEB, Beijing, China) was used to digest the template plasmid.

## Figures and Tables

**Table 1:** Primers used in this study.

Primer	Sequence (5′to 3′)
CS-*Rspo3*-ORF-Fw	TGGGCTACTATGCAATTACAACTG
CS-*Rspo3*-ORF-Rv	AGAGGGAGCTCACTGTACAA
CS-*Rspo3*-WISH-Fw	ATTTAGGTGACACTATAGAAGNGGGCACCGGCAACATAAAC
CS-*Rspo3*-WISH-Rv	TAATACGACTCACTATAGGGAGACTCCACTCACCCACTTCAC
CS-*Rspo3*-RT-PCR-Fw	GGGTCCTGTTGTGTTTAGGA
CS-*Rspo3*-RT-PCR-Rv	TGCTGACCACCAGTGTAATC
CS-*Rspo3*-mRNA-Fw	TAATACGACTCACTATAGGGAGAATGCAATTACAACTGATCTCCTTTG
CS-*Rspo3*-mRNA-Rv	AGAGGGAGCTCACTGTACAA
CS-*Rspo3*-ORF-EC-Fw	CGCGGATCCGCCACCATGCAATTACAACTGATCTCC
CS-*Rspo3*-ORF-EC-Rv	CCGGAATTCGCTGTACAAGGTCATCATC
CS-*Rspo3*-delFU1-Fw	CGGCAACATAAACAGATCATGCGCTCTCACGAAAGA
CS-*Rspo3*-delFU1-Rv	TCTTTCGTGAGAGCGCATGATCTGTTTATGTTGCCG
CS-*Rspo3*-delFU2-Fw	CCTCTGTGCGTCACTTTCGTGAGAGCGCATGC
CS-*Rspo3*-delFU2-Rv	GCATGCGCTCTCACGAAAGTGACGCACCAGAGG
CS-*Rspo3*-delFU3-Fw	GGTCCGCAGTGACGCAAACGACAAACTCATGG
CS-*Rspo3*-delFU3-Rv	CCATGAGTTTGTCGTTTGCGTCACTGCGGACC
CS-*Rspo3*-delTSP-Fw	CCACAAGTGCACTGTGAAGGACGGAGGAAGAATG
CS*-Rspo3*-delTSP-Rv	CATTCTTCCTCCHTCCTTCACAGTGCACTTGTGG
B2M-RT-PCR-Fw	TGTTCGTCGTTC TGCCGTGT
B2M-RT-PCR-Rv	TCAGGGTGTTGGGCTTGTTGT
DR-VEGFa-RT-PCR-Fw	AGTTATTTCTCGCGGCTCTCC
DR-VEGFa-RT-PCR-Rv	ACACATCCATGAAGGGAATCAC
DR-*ntl*-RT-PCR-Fw	GGATGAAAGCACCCGTATC
DR-*ntl*-RT-PCR-Rv	GTGTATCCTGGGTTCGTATTT
DR-*boz*-RT-PCR-Fw	TAGAGACAGAGCAAGAGGAG
DR-*boz*-RT-PCR-Rv	GGTGTCTCCTAAGATGTAATCAA
DR-ZNRF3-RT-PCR-Fw	TTGGACCCAAGCTGTCTTAC
DR-ZNRF3-RT-PCR-Rv	CACGCTGACCCTTGAACTTA
DR-*sp5l*-RT-PCR-Fw	GGAGGTCACGTTGAGGATGG
DR-*sp5l*-RT-PCR-Rv	GCGACAGCGACGAGTAGAGC
DR-*β-actin*-RT-PCR-Fw	CCCAGACATCAGGGAGTGAT
DR-*β-actin*-RT-PCR-Rv	TCTCTGTTGGCTTTGGATT
DR-*ntl*-WISH-Fw	ATTTAGGTGACACTATAGAAGNGATGACTTCTTCCAACCCCG
DR-*ntl*-WISH-Rv	TAATACGACTCACTATAGGGAGATCAGTAGCTCTGAGCCACAGG
